# Supplementary material for: In vivo topology converts competition for cell-matrix adhesion into directional migration
Source: Nat Commun. 2019 Apr 3;10:1518. doi: 10.1038/s41467-019-09548-5 (PMC6447549; doi:10.1038/s41467-019-09548-5)
Supplement: Supplementary file 1 — Supplementary Information [file 41467_2019_9548_MOESM1_ESM.pdf]

SUPPLEMENTARY INFORMATION

In vivo topology converts competition for cell-matrix adhesion into directional migration

Bajanca et al.

Supplementary Figures

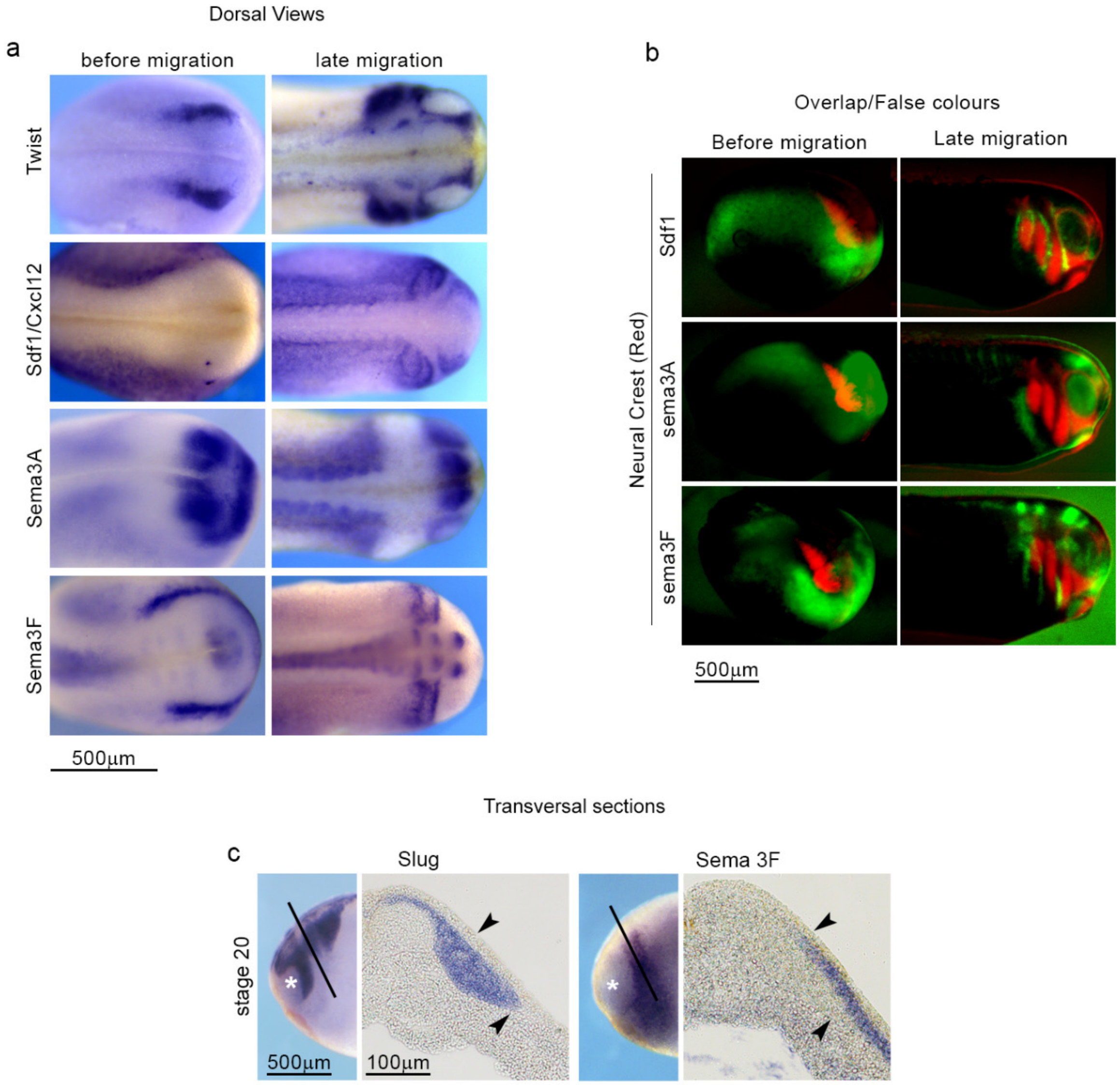

Supplementary figure 1. Distribution of neural crest with respect to Sdf1, Sema3A and 3F expression at early and late stages of migration. (a) Dorsal views of in situ hybridization for Twist (NC marker), Sdf1, Sema3A and Sema3F at premigration (st17) and late migration stages (st27/28). (b) Overlay of in situ images shown in Figure 1a after conversion in false colours. (c) Transversal sections at stage 20 (early migration) showing that NC cells migrate directly underneath the Sema-positive ectoderm violating the Sema-/Sema+ boundary.

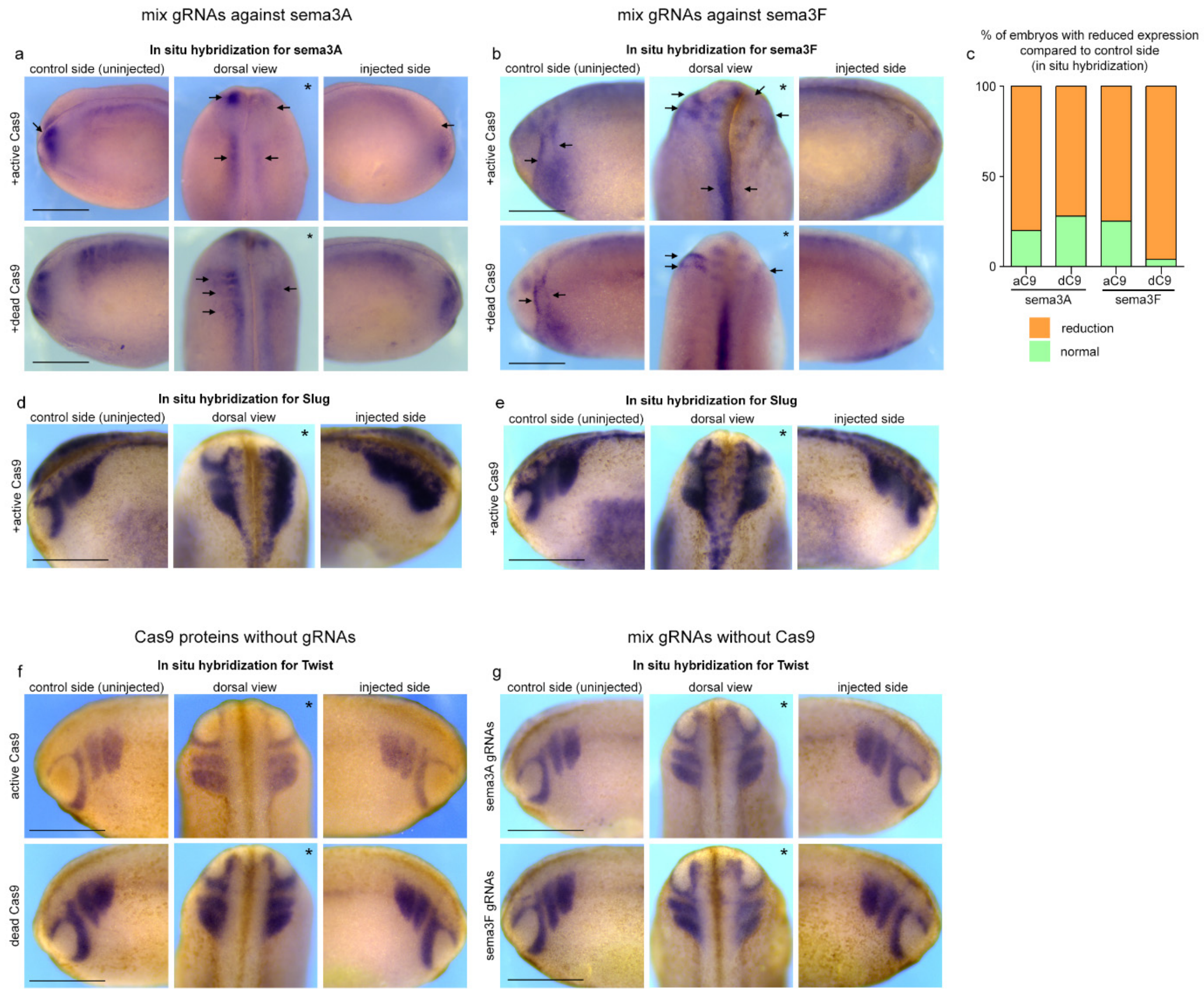

Supplementary figure 2. CRISPR/Cas9-driven knockdowns specifically reduce the expression of Sema3A and 3F.

(a) In situ hybridization against Sema3A after co-injection of Sema3A gRNAs and active Cas9 (top row, n=35) or after co-injection of Sema3A gRNAs and dead Cas9 (bottom row, n=11). (b) In situ hybridization against Sema3F after co-injection of Sema3F gRNAs and active Cas9 (top row, n=12) or after co-injection of Sema3F gRNAs and dead Cas9 (bottom row, n=24). (c) Percentage of embryos with normal (green) or reduced (orange) expression of Sema3A and 3F with each Cas9-based knockdown. N=82 embryos from 2 independent experiments. Arrows point to regions of interest where the downregulation of Sema3A/3F is clearly visible. (d-e) in situ hybridization against Slug after knockdown of sema3A (d, n=6) or sema3F (e, n=7) confirming that neural crest cells are properly induced and showing that CRISPR/Cas9 does not have a general non-specific effect on gene expression. (f) In situ hybridization against Twist after injection of active Cas9 (top row, n=11) or dead Cas9 (bottom row, n=17) without gRNAs. Note that neural crest distribution is not affected. (g) In situ hybridization against Twist after injection of the mix of gRNAs against sema3A (top row, n=14) or sema3F (bottom row, n=16). Note that neural crest distribution is not affected. Asterisks indicate the injected side. Scale bars, 500 microns.

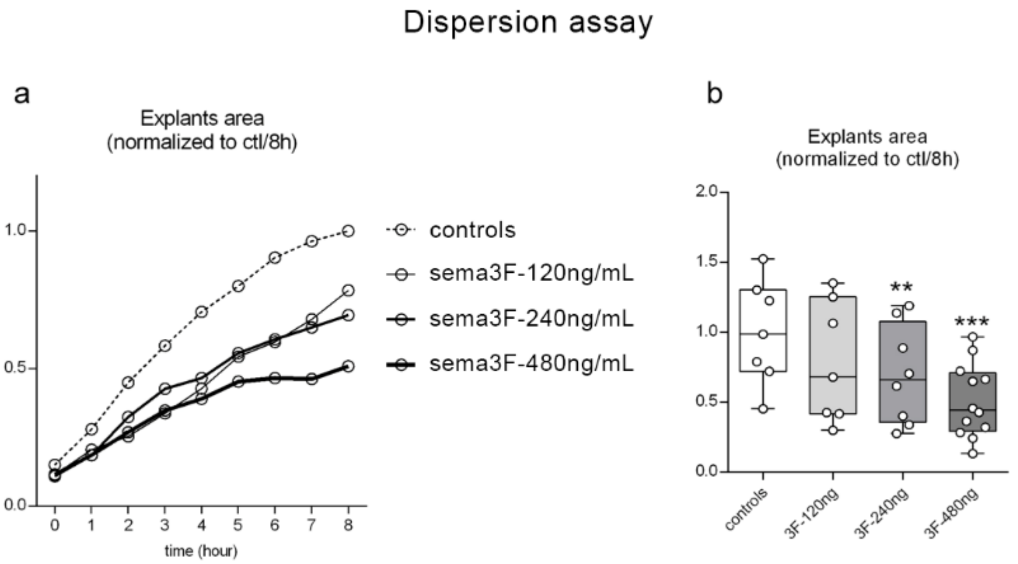

Supplementary figure 3. Semaphorin 3F inhibits dispersion of NC explants in a dose-dependent manner.

(a) Normalized explants area overtime. (b) Distribution of explants area after 8 hours of migration for each experimental condition. (c) Stills from time-lapse movies for each experimental condition. N= 34 explants from 2 independent experiments. ANOVA followed by multiple comparisons, **, p=0.0247; ***, p=0.0001. Box and whiskers plot: the box extends from the 25th to the 75th percentile; the whiskers show the extent of the whole dataset. The median is plotted as a line inside the box.

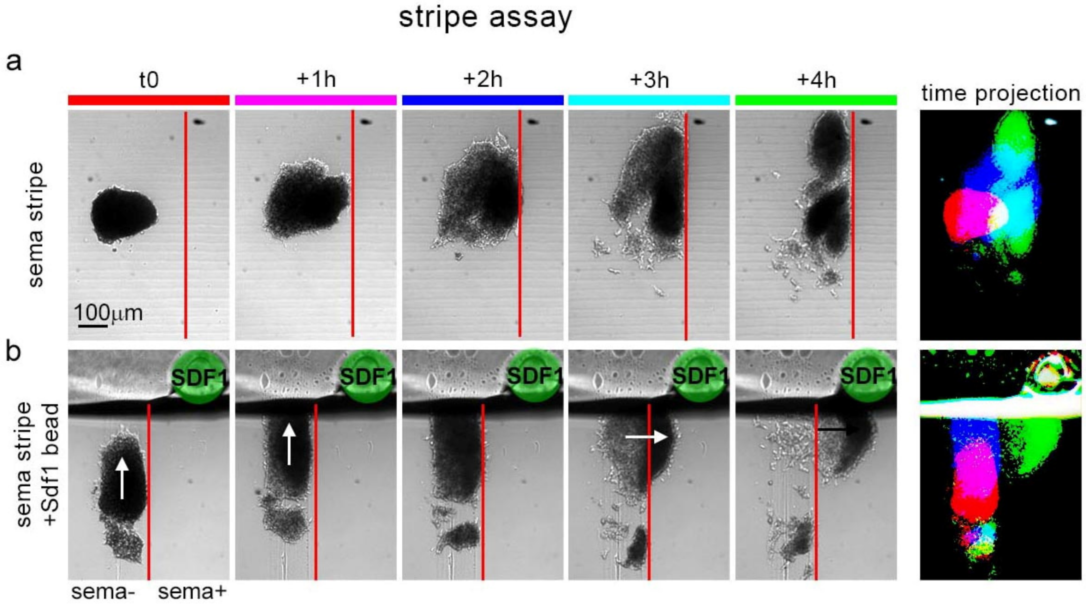

Supplementary figure 4. Sdf1 is sufficient to attract NC cells into Sema3A-positive domains.
(a) Control NC cells cultured on a Sema-negative stripe of Fibronectin. (b) Control NC cells cultured on a Sema-negative stripe of Fibronectin, exposed to an Sdf1-soaked bead. Note that NC cells are first attracted to Sdf1 while remaining on the Sema-negative stripe. When NC cells have reached the vicinity of the Sdf1 source, they cross the Sema-/Sema+ boundary. $N_{explants}$ on Sema stripe=19, $N_{explants}$ on Sema+Sdf1=7, from two independent experiments.

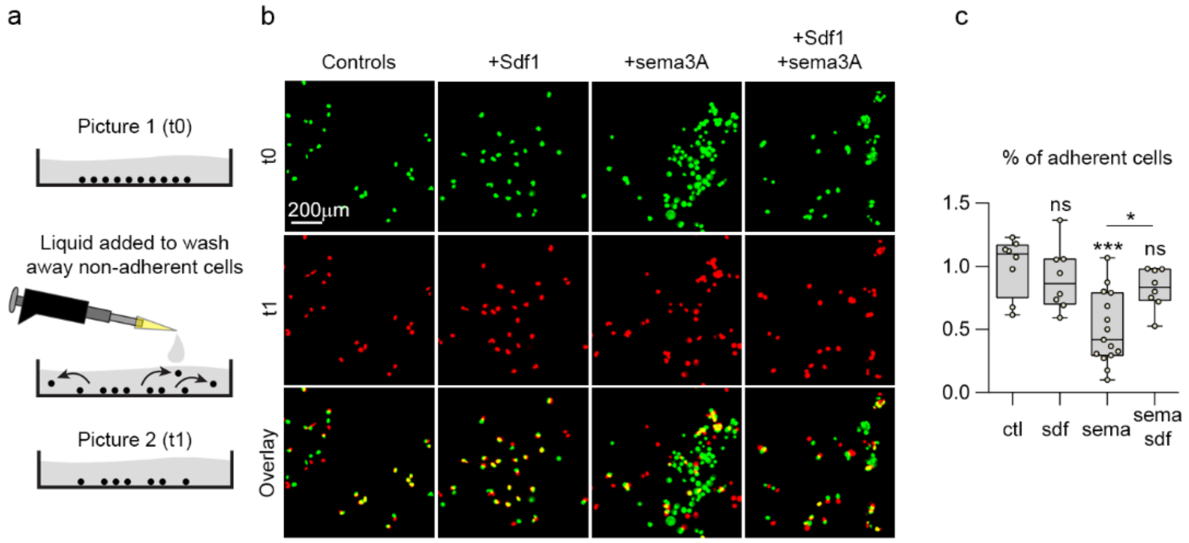

Supplementary Figure 5. Sdf1 rescues Sema3A's effect on cell adhesion.
(a) Cells extracted from Xenopus embryos at stage 18 are dissociated and plated on Fibronectin. A picture is taken. Culture medium is then added to wash away non-adherent cells before a second picture is taken. (b) Images of cells under each experimental condition before and after washing. (c) Percentage of adherent cells in each condition. ANOVA, followed by multiple comparisons; ***, p=0.0002; *, p=0.0207. Box and whiskers plot: the box extends from the 25th to the 75th percentile; the whiskers show the extent of the whole dataset. The median is plotted as a line inside the box.

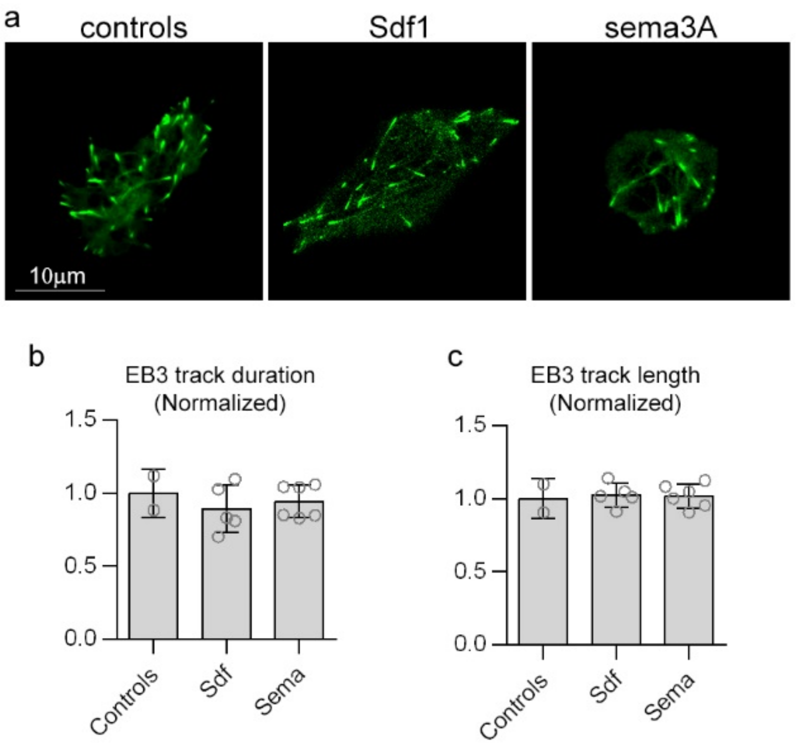

Supplementary figure 6. Dissociated NC cells transfected with EB3-GFP.
(a) Stills from time-lapse movies showing control NC cells (left) and cells exposed to Sema3A (right). (b) Average EB3 track length cells in control condition or exposed to Sdf1 or Sema3A. (c) Average track duration for cells in control condition or exposed to Sdf1 or Sema3A. N_{exp}=1, n_{cells}= 13, n_{tracks}= 4663. Error bars, standard deviation. Each dot represents mean value of the measured parameter per single cell analyzed.

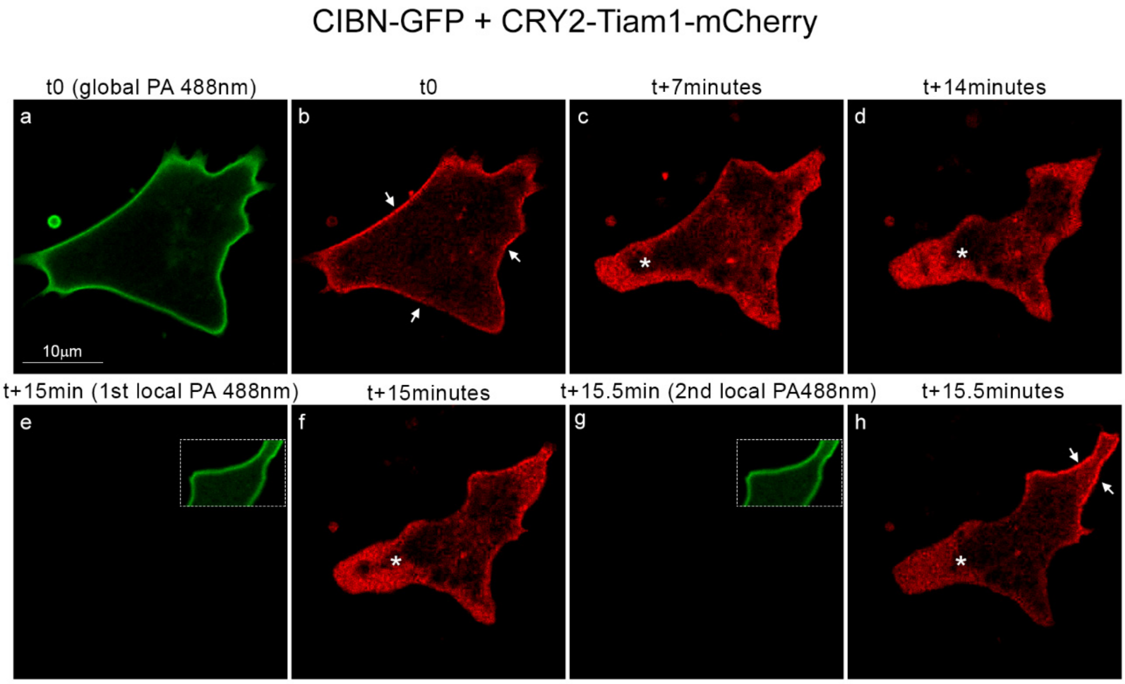

Supplementary figure 7. Validation of light-dependent membrane localization of CRY2-Tiam1-mCherry in cells co-transfected with CIBN-CaaX-GFP in Xenopus NC cells.

(a) Single NC cells after global illumination at 488nm, CIBN-GFP. (b) Membrane localization of Tiam1-CRY2-mCherry after global illumination (arrows). (c-d) Progressive diffusion of Tiam1 into the cytoplasm (asterisks) after illumination was turned off. (e) First round of local illumination at 488nm. (f) Tiam1 localization after local illumination. Note that Tiam1 is located in the cell membrane where the blue light was shone, whereas it is still cytoplasmic (asterisk) elsewhere. (g-h) Second round of local illumination at 488nm. Note that membrane localization is reinforced (h, arrows) by this second photoactivation while in other parts of the cell Tiam1 kept diffusing into the cytoplasm (asterisk). PA, photoactivation.

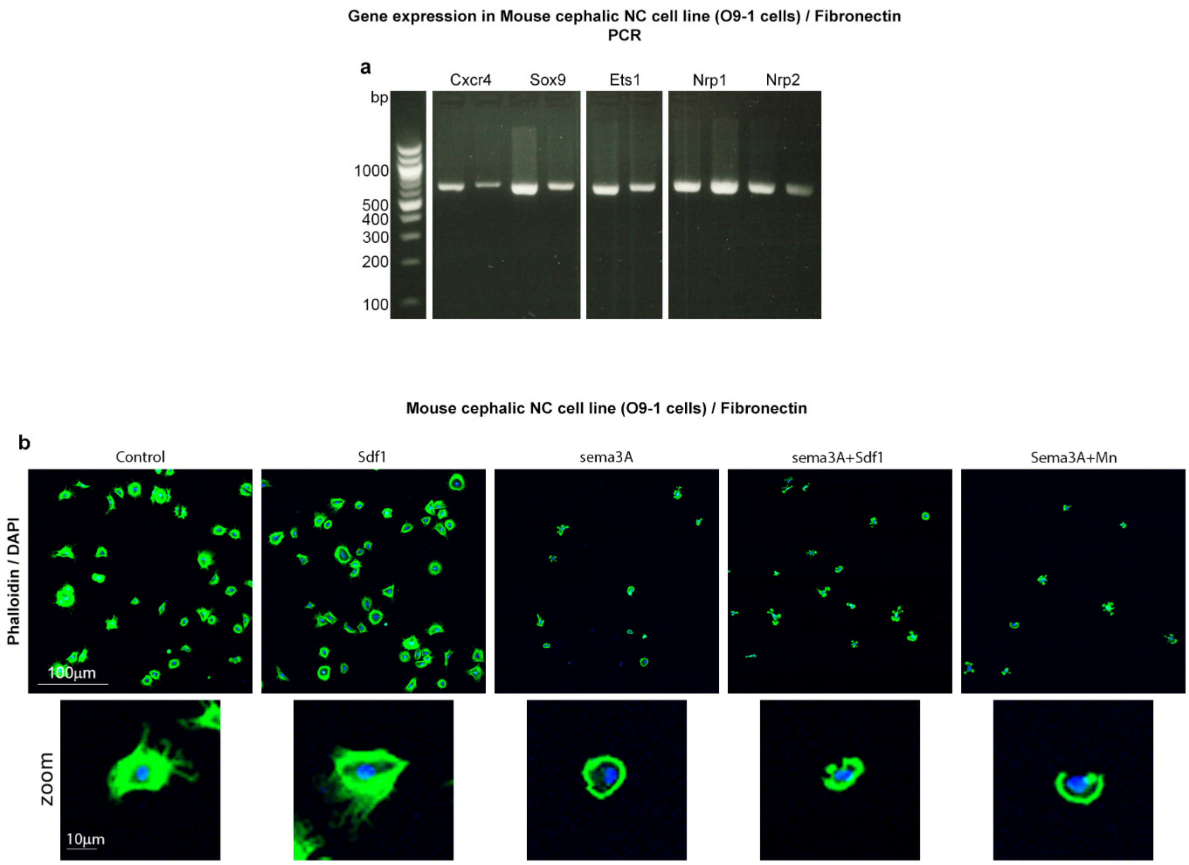

Supplementary figure 8. Mouse NC cells, O9-1, cultured on Fibronectin.
(a) PCR results showing that O9-1 cells express Cxcr4, Nrp1 and Nrp2, two samples from independent cultures were run in parallel for each target gene. Original gel image is provided in the Source Data File. (b) Low magnification images of O9-1 cells cultured on Fibronectin with, from left to right: control medium, sdf1 added in solution (0.5µg/mL), Sema3A coated at 60ng/mL, Sema3A and Sdf1 together, Sema3A and Mn2+ added at 2mM final. (c) Zooms on representative cells for each condition shown in (a). n= 4368 cells.
