# Supplementary material for: In vivo topology converts competition for cell-matrix adhesion into directional migration
Source: Nat Commun. 2019 Apr 3;10:1518. doi: 10.1038/s41467-019-09548-5 (PMC6447549; doi:10.1038/s41467-019-09548-5)
Supplement: Supplementary file 3 — Reporting Summary [file 41467_2019_9548_MOESM3_ESM.pdf]

nature research

Corresponding author(s): ERIC THEVENEAU

Last updated by author(s): Feb 4, 2019

Reporting Summary

Nature Research wishes to improve the reproducibility of the work that we publish. This form provides structure for consistency and transparency in reporting. For further information on Nature Research policies, see Authors & Referees and the Editorial Policy Checklist.

Statistics

For all statistical analyses, confirm that the following items are present in the figure legend, table legend, main text, or Methods section.

n/a	Confirmed	
☐	☒	The exact sample size (*n*) for each experimental group/condition, given as a discrete number and unit of measurement
☐	☒	A statement on whether measurements were taken from distinct samples or whether the same sample was measured repeatedly
☐	☒	The statistical test(s) used AND whether they are one- or two-sided<br>*Only common tests should be described solely by name; describe more complex techniques in the Methods section.*
☐	☒	A description of all covariates tested
☐	☒	A description of any assumptions or corrections, such as tests of normality and adjustment for multiple comparisons
☐	☒	A full description of the statistical parameters including central tendency (e.g. means) or other basic estimates (e.g. regression coefficient) AND variation (e.g. standard deviation) or associated estimates of uncertainty (e.g. confidence intervals)
☐	☒	For null hypothesis testing, the test statistic (e.g. *F*, *t*, *r*) with confidence intervals, effect sizes, degrees of freedom and *P* value noted<br>*Give P values as exact values whenever suitable.*
☒	☐	For Bayesian analysis, information on the choice of priors and Markov chain Monte Carlo settings
☒	☐	For hierarchical and complex designs, identification of the appropriate level for tests and full reporting of outcomes
☒	☐	Estimates of effect sizes (e.g. Cohen's *d*, Pearson's *r*), indicating how they were calculated

Our web collection on statistics for biologists contains articles on many of the points above.

Software and code

Policy information about availability of computer code

Data collection	Leica LAS X, Zeiss ZEN, Fiji, Imaris 7.2, GraphPad Prism 6
Data analysis	Fiji, Imaris 7.2, GraphPad Prism 6

For manuscripts utilizing custom algorithms or software that are central to the research but not yet described in published literature, software must be made available to editors/reviewers. We strongly encourage code deposition in a community repository (e.g. GitHub). See the Nature Research guidelines for submitting code & software for further information.

Data

Policy information about availability of data

All manuscripts must include a data availability statement. This statement should provide the following information, where applicable:

- Accession codes, unique identifiers, or web links for publicly available datasets
- A list of figures that have associated raw data
- A description of any restrictions on data availability

All relevant data are available from the authors upon reasonable request.

Field-specific reporting

Please select the one below that is the best fit for your research. If you are not sure, read the appropriate sections before making your selection.

☒ Life sciences ☐ Behavioural & social sciences ☐ Ecological, evolutionary & environmental sciences

For a reference copy of the document with all sections, see nature.com/documents/nr-reporting-summary-flat.pdf

Life sciences study design

All studies must disclose on these points even when the disclosure is negative.

Sample size	We analyzed the whole population of embryos available in each experiments excluding non viable embryos
Data exclusions	For large datasets automated search for outliers was performed using GraphPad Prims6
Replication	Each experiments is described with its N of independent experiments and n samples used in the figure legends
Randomization	The whole population of embryos available for each experiment was divided at random
Blinding	Blinding was not used. Non viable embryos/cells need to be excluded in each experiments prior to processing and analyses.

Reporting for specific materials, systems and methods

We require information from authors about some types of materials, experimental systems and methods used in many studies. Here, indicate whether each material, system or method listed is relevant to your study. If you are not sure if a list item applies to your research, read the appropriate section before selecting a response.

Materials & experimental systems

n/a	Involved in the study	
☐	☒	Antibodies
☐	☒	Eukaryotic cell lines
☒	☐	Palaeontology
☐	☒	Animals and other organisms
☒	☐	Human research participants
☒	☐	Clinical data

Methods

n/a	Involved in the study	
☒	☐	ChIP-seq
☒	☐	Flow cytometry
☒	☐	MRI-based neuroimaging

Antibodies

Antibodies used	rabbit anti-Phospho-Paxillin Tyr118 (Upstate, 07-733), mouse anti-Rac1-GTP (NewEast, 26903). Secondary antibodies: goat anti-rabbit Alexa-488 or 555, goat anti-mouse IgM-594 (Invitrogen).
Validation	rabbit anti-Phospho-Paxillin Tyr118 (Upstate, 07-733), validated in Kuriyama et al Journal of Cell Biology, 2014.<br>mouse anti-Rac1-GTP (NewEast, 26903), validated in Cancer Lett. 2015 Jan 28;356(2 Pt B):382-91. doi: 10.1016/j.canlet.2014.09.030.

Eukaryotic cell lines

Policy information about cell lines

Cell line source(s)	O9-1 Mouse cranial neural crest cell line Millipore (SCC049)
Authentication	We checked that these cells were expressing Sox9 and Ets1 but not Sox10 as initially described in Ishii et al Stem Cells Dev. 2012 Nov 20;21(17):3069-80. doi: 10.1089/scd.2012.0155.
Mycoplasma contamination	Not tested for mycoplasma contamination
Commonly misidentified lines (See ICLAC register)	n.a.

Animals and other organisms

Policy information about studies involving animals; ARRIVE guidelines recommended for reporting animal research

Laboratory animals	Xenopus laevis
Wild animals	n.a.
Field-collected samples	n.a
Ethics oversight	Animal care and experimentation were conducted in accordance with institutional and national guidelines, under the

Ethics oversight	institutional licence number A 31 555 01 delivered by the Préfecture de la Haute-Garonne, running until March 2019.

Note that full information on the approval of the study protocol must also be provided in the manuscript.
