# Supplementary material for: In vivo topology converts competition for cell-matrix adhesion into directional migration
Source: Nat Commun. 2019 Apr 3;10:1518. doi: 10.1038/s41467-019-09548-5 (PMC6447549; doi:10.1038/s41467-019-09548-5)
Supplement: Supplementary file 4 — Description of Additional Supplementary Files [file 41467_2019_9548_MOESM4_ESM.pdf]

Description of Additional Supplementary Files

File Name: Supplementary Movie 1
Description: In vivo NC cell migration is impaired in sema3A-KD environments. Top left, control NC cells grafted into a host embryo injected with control MO. Top right, control NC cells grafted into a host embryo injected with Sema3A-MO. Bottom left, control NC cells grafted into a host embryo injected with Sema3A-gRNA + active Cas9. Bottom right, control NC cells grafted into a host embryo injected with Sema3A-gRNA + dead Cas9. 1 picture every 10 minutes, 6 hours. 5X objective.

File Name: Supplementary Movie 2
Description: Sema3A impairs dispersion of NC explants on Fibronectin From left to right: Fibronectin, Fibronectin plus Sema3A coated at 15, 30 and 60ng/mL. Two examples for each condition. 1 picture every 3 minutes, 8 hours. 10X objective.

File Name: Supplementary Movie 3
Description: Dispersion of NC explants under Sema3A conditions is rescued by Sdf1 Two top rows, from left to right: Fibronectin, Fibronectin plus Sema3A coated at 15, 30 and 60ng/mL. Two examples for each condition. Two bottom rows from left to right: same conditions as above plus Sdf1 added in solution at 0.5µg/mL. Two examples for each condition. 1 picture every 3 minutes, 8 hours. 10X objective.

File Name: Supplementary Movie 4
Description: Sema3F impairs dispersion of NC explants on Fibronectin Left column, NC explants on Fibronectin, two examples. Middle and right column, NC explants on Fibronectin plus Sema3F coated at 480ng/mL, four examples. 1 picture every 3 minutes, 8 hours. 10X objective.

File Name: Supplementary Movie 5
Description: Sdf1 attracts NC cells into Sema3A-positive domain part 1. Top row, NC explants cultured on a Fibronectin stripe next to a Fibronectin plus Sema3A stripe. Middle row, NC explants cultured on a Fibronectin stripe next to a Fibronectin plus Sema3A stripe in which a bead soaked in PBS was placed. Bottom row: NC explants cultured on a Fibronectin stripe next to a Fibronectin plus Sema3A stripe in which a bead soaked in Sdf1 was placed. The same sequence is played twice. Green asterisks indicate regions were NC cells violate the Sema-/Sema+ boundary to reach Sdf1. Movie acquired on a dissecting microscope. Dissecting microscope, 1 picture every 10 minutes. Nctl= 4 explants, NPBS= 4 explants, NSdf1= 5 explants.

File Name: Supplementary Movie 6
Description: Sdf1 attracts NC cells into Sema3A-positive domain part 2. Three examples of NC explants cultured on a Fibronectin stripe next to a Fibronectin plus Sema3A stripe in which a bead soaked in Sdf1 was placed, immobilized by silicone grease. Note that cells move in two distinct phases. First, there is directional migration towards Sdf1. Then, NC cells migrate into the Semaphorin domain. 1 picture every 5 minutes, 10X objective.

File Name: Supplementary Movie 7
Description: Sdf1 attracts NC cells into Sema3A-positive domain part 3. Left panel, NC cells cultured on a Fibronectin stripe next to a Fibronectin plus Sema3A stripe. Right panel, NC cells cultured on a Fibronectin stripe next to a Fibronectin plus Sema3A stripe in which a bead soaked in Sdf1 was placed. Epifluorescence microscope, 20X objective, 1 picture every 5 minutes.

File Name: Supplementary Movie 8
Description: Sema3A affects cell spreading and protrusive activity. Cells were transfected with Life-Act-GFP. Top left panel, Fibronectin. Top right panel, Sdf1 added in solution. Bottom left panel, Fibronectin plus coated Sema3A. Bottom right panel, Fibronectin plus coated Sema3A plus Sdf1 added in solution. Confocal microscope, 40X objective, 1 picture 5 seconds, duration, 8 minutes.

File Name: Supplementary Movie 9
Description: Sema3A does not affect microtubule polymerization. Cells were transfected with EB3-GFP. Left, Fibronectin. Middle, Sdf1 added in solution. Right, Fibronectin plus coated Sema3A. Confocal microscope, 40X objective, 1 picture every 2 seconds, duration, 4 minutes.

File Name: Supplementary Movie 10
Description: Photoactivation of Tiam1 allows NC cells cultured on Sema3A to form protrusions. Columns from left to right: CRY2-Tiam1-mCherry, CIBN-CaaX-GFP, merge. Top row: cells transfected with CRY2- Tiam1-mCherry and CIBN-CaaX-GFP cultured on Fibronectin. Note that these cells can form protrusions even when the light is off. Middle and bottom row, two examples of cells transfected with CRY2-Tiam1-mCherry, CIBN-CaaX-GFP cultured on Fibronectin plus Sema3A. Note that these cells become round when the light is off and start spreading when the light is turned on. 40X objective, 1 picture every minute.

File Name: Supplementary Movie 11
Description: Sustained photoillumination rescues Sema3A's effects in cells expressing CRY2-Tiam1 and CIBN but not in cells expressing CIBN only. Left column, cells transfected with CRY2-Tiam1-mCherry and CIBN-CaaX-GFP cultured on Fibronectin. Middle columns, cells

transfected with CIBN-CaaX-GFP only cultured on Fibronectin plus Sema3A. Right column: cells transfected with CRY2-Tiam1-mCherry and CIBN-CaaX-GFP cultured on Fibronectin plus Sema3A. 40X objective, 1 picture every 1 minute.

File Name: Supplementary Movie 12
Description: Dispersion of NC explants under Sema3A conditions is rescued by Mn2+. Left column: NC explants cultured on Fibronectin. Other three columns NC explants cultured on Fibronectin plus Sema3A with, from left to right, control culture medium, Mn2+ added in solution (2mM) and Mn (2mM) plus CuE (1nM) added in solution, respectively. Three examples of each experimental condition. 10X objective, 1 picture every 3 minutes. Duration 8 hours.

File Name: Supplementary Movie 13
Description: Global unpolarised photoactivation of Tiam1 rescues the in vivo dorso-ventral migration of Cxcr4MO-injected NC cells. Left column, two examples of NC cells co-injected with Cxcr4MO and CIBN-GFP grafted into a control host. Right column, two examples of NC cells co-injected with Cxcr4MO, CIBN-GFP and PA-Tiam1. 1 picture every 10 minutes, 6 hours. 5X objective.
